# Percutaneous closure of ventricular septal rupture after myocardial infarction: A retrospective study of 81 cases

**DOI:** 10.1002/clc.24027

**Published:** 2023-05-15

**Authors:** Tongfeng Chen, Yuhao Liu, Jing Zhang, Zirui Sun, Yu Han, Chuanyu Gao

**Affiliations:** ^1^ Henan Key Laboratory of Coronary Heart Disease Control, Heart Center of Henan Provincial People's Hospital, Henan Research Center for Cardiovascular Epidemiology, Central China Fuwai Hospital People's Hospital of Zhengzhou University Zhengzhou China

**Keywords:** acute myocardial infarction, percutaneous closure, ventricular septal rupture

## Abstract

**Objective:**

To investigate the efficacy and safety of percutaneous closure of ventricular septal rupture (VSR) after acute myocardial infarction (AMI).

**Methods:**

This retrospective study included 81 patients who underwent transcatheter closure for postinfarction VSR. We analyzed clinical data from hospitalization and the 30‐day follow‐up, compared clinical data from the survival and death groups, and explored the best closure time and the safety and efficacy of occlusion. The risk factors for death at 30 days were analyzed by logistic regression.

**Results:**

C‐reactive protein (CRP), white blood cell counts, N‐terminal pro brain natriuretic peptide (NT‐ProBNP), and aspartate aminotransferase were higher in the death group than in the survival group (*p* < .01), with a higher rate of application of vasoactive drugs, and a shorter time from AMI to operation (*p* < .05). At 30 days postocclusion, 19 patients (23.5%) had died. The mortality rate was significantly lower for operation performed 3 weeks after AMI than for operation performed within 3 weeks of AMI (12.5% vs. 48%, *p* < .001). Devices were successfully implanted in 76 patients, with 16 (21.1%) operation‐related complications and 12 (15.8%) valve injuries. Cardiac function improved significantly (*p* < .001) at discharge (*N* = 66) and 30 days after procedure (*N* = 62). Qp/Qs and pulmonary artery systolic pressure decreased significantly, while aortic systolic pressure increased significantly (*p* < .001). Additionally, EF and LVDd improved (*p* < .05) after occlusion. Increases in CRP and NT‐ProBNP were risk factors for death at 30 days after closure (*p* < .05).

**Conclusion:**

Percutaneous VSR closure can be a valuable treatment option for suitable patients with VSR.

## Graphical abstract

Ventricular septal rupture (VSR) is a rare but life‐threatening complication of acute myocardial infarction (AMI). In this retrospective study of 81 patients, we investigated the efficacy and safety of percutaneous closure of postinfarction VSR. Our results show that delayed closure (3 weeks after AMI) is associated with a lower mortality rate and better clinical outcomes. We also found that percutaneous VSR closure can significantly improve cardiac function and hemodynamics. Notably, we identified that C‐reactive protein and N‐terminal pro brain natriuretic peptide elevation are independent risk factors for death at 30 days after VSR closure. Our study highlights the value of percutaneous closure as a viable treatment option for suitable VSR patients.

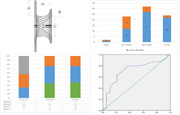

## 1 INTRODUCTION

1.1

Ventricular septal rupture (VSR) is a rare mechanical complication after acute myocardial infarction (AMI), occurring in approximately 0.26% of patients with acute coronary syndromes.^1^ It has a high mortality rate in the early stages, approximately 67%−82% within 1 week of conservative treatment with drugs, and a 1‐year survival rate of only 7%.^2^, ^3^ Surgical repair combined with coronary artery bypass grafting was previously used to manage patients with VSR, but there are limitations, such as a high perioperative mortality rate.^4^ For patients with AMI complicated with VSR, surgical treatment might lead to a 30‐day mortality rate of up to 47%.^5^ With advances in interventional technology, percutaneous VSR closure has attracted increasing attention. To date, few studies have reported such interventional strategies, and large‐scale clinical trials are lacking. This study reports 81 consecutive AMI patients who underwent percutaneous VSR closure in our center from October 2013 to May 2020. The report is as follows.

## 2 MATERIALS AND METHODS

### 2.1 Population (study cohort)

This was a retrospective analysis of 81 patients who underwent interventional percutaneous VSR closure in the Department of Cardiovascular Medicine of Henan Provincial People's Hospital and Fuwai Central China Cardiovascular Hospital from October 2013 to May 2020. There were 76 successful cases and 5 failures. Clinical data on hospitalization were collected. Patients who survived to discharge were scheduled for a 30‐day follow‐up by telephone interview.

The inclusion criteria were as follows. (1) AMI was diagnosed in accordance with the Universal Definition of Myocardial Infarction (fourth edited),^6^ and the presence of VSR was observed on ultrasonography or ventriculography after AMI. (2) Informed consent was obtained from the patient/legal guardian.

The exclusion criteria were as follows. (1) Informed consent was not available from the patient or legal guardian, and the patient refused to comply with treatment or follow‐up. (2) The defect had a diameter >22 mm or was very close to structures such as the valve or chordae tendineae, which was not suitable for closure. (3) The patient's cardiac function was not effectively controlled, and the patient was unable to maintain a supine position for more than half an hour. (4) The patient had other comorbid diseases with an expected lifetime of less than 1 year. (5) The patient had a history of infective endocarditis in the previous 3 months. (6) The patient had coagulation dysfunction that had not been effectively controlled.

The Ethics Committee of Fuwai Central China Cardiovascular Hospital provided approval for the study.

### 2.2 Interventional procedure

Basic procedures referred to the common view of Chinese medical experts on interventional treatment of ventricular septal defects.^7^ Procedure steps: Deliver a 6 F pigtail catheter into the left ventricle near the apex, then perform left ventriculography at a left anterior oblique position of 35−50° and a cephalad position of 10° to assess the morphology, location, and diameter of the defect. Use a pigtail catheter at the cutting end or a JR 4.0 contrast catheter to assist in passing a super‐slip guidewire through the defect to establish an arteriovenous track. Confirm the track is smooth under digital subtraction angiography to avoid loop tendon cords. Deliver the delivery sheath to the left heart system along the venous side of the track and release the occluder after it has been delivered along the delivery sheath to the appropriate position. Perform left ventriculography again to confirm that the occluder is fixed in position and well blocked, and then release the occluder. The closure device (A7B3H10) used here was from Shanghai Shape Memory Alloy Co. Ltd., as shown in Supporting Information: Figure 1, the closure device with a 7 mm left ventricular side rim (A), a 3 mm right ventricular side rim (B), and a 10 mm waist height (H). The diameter of the occluder was 8−14 mm larger than the perforation diameter, according to the size or the myocardial tissue weakness around the defect.

### 2.3 Outcome measures

General clinical data on the hospitalization of 81 patients who underwent attempted interventional percutaneous VSR closure were collected. Patients who survived to discharge (*n* = 66) were followed up for 30 days. Primary safety outcomes: closure failure and all‐cause mortality at 30 days after closure. Secondary safety outcomes: procedural complications, such as valve damage, vascular injury, hemolysis, and high‐degree atrioventricular block; other complications independent of occlusion, such as major adverse cardiovascular events (MACEs), stroke, worsening of heart failure, peripheral organ failure, and bleeding. Effectiveness outcomes: improvement in cardiac function, pulmonary/systemic flow ratio (Qp/Qs), pulmonary artery systolic pressure (PASP), aortic systolic pressure (ASP), left ventricular ejection fraction (LVEF), and left ventricular internal diameter at end‐diastole (LViDd).

Criteria for valve damage: (1) presence of newly developed rupture of the chordae tendineae or prolapse of the valve or blockade of the valve by the occluder on ultrasound; (2) presence of severe postoperative valve regurgitation, with mild/no valve regurgitation before the operation; and (3) presence of severe postoperative valve regurgitation, with moderate valve regurgitation before the operation, along with the regurgitation area increased by more than 5 cm^2^. Valve damage was defined as the occurrence of the above conditions.

### 2.4 Statistical analysis

SPSS 22.0 was used to perform the statistical analysis. Measurement data that conformed to a normal distribution were represented by mean ± standard deviation and then analyzed with an independent sample *t*‐tests for between‐group comparisons or a paired sample *t*‐test for within‐group comparisons. For data with a skewed distribution, the data were expressed as the median (interquartile range) and analyzed with the *U* test. Comparisons of categorical variables in percent (%) were performed with the *χ* ^2^ test. Multivariate logistic regression analysis was conducted. A *p* value less than or equal to .05 was considered to indicate a statistically significant difference.

## 3 RESULTS

### 3.1 General clinical information

There were 81 VSR cases with percutaneous closure, including 76 successful cases (93.8%) and 16 deaths at the 30‐day follow‐up. There were 47 (58%) females with an average age of 67 (40−80) years. Patients who were discharged from the hospital were followed‐up for 30 days and divided into a survival group and a death group according to the outcome. The general clinical data of the 81 patients are shown in Table 1.

**Table 1:** General clinical information.

Clinical outcomes	Patients with interventional treatment (*N* = 81)	Survival (*N* = 62)	Death (*N* = 19)	*p* Value
Age (years)	67 (63−71)	67 (62.5−71.25)	67 (65−71)	.604
Female (%)	47 (58%)	36 (58.1%)	11 (57.9%)	.990
History of coronary heart disease (%)	11 (3.6%)	8 (12.9%)	3 (15.8%)	.748
Hypertension (%)	47 (58%)	37 (59.7%)	10 (52.6%)	.586
Diabetes (%)	24 (29.6%)	18 (29%)	6 (31.6%)	.832
Cerebral infarction (%)	17 (21%)	11 (17.7%)	6 (31.6%)	.195
Hyperlipidemia (%)	11 (13.6%)	8 (12.9%)	3 (15.8%)	.748
Admission HR (beat/min)	93.5 ± 18.8	92 ± 19.2	98.6 ± 17.0	.184
Admission SBP (mmHg)	108.8 ± 15.8	108.8 ± 15.2	104.4 ± 17.7	.292
Admission LVEF (%)	53 (45−56)	53 (45.75−56.25)	46 (44.25−55.75)	.257
Admission PASP (mmHg)	54.3 ± 14.2	53.6 ± 14.6	56.8 ± 13	.390
Admission LViDd (mm)	53.4 ± 5.8	53.7 ± 5.9	52.6 ± 5.6	.481
CRP (mg/L)	15.8 (5.7−45.5)	10.5 (3.74−34.17)	44.1 (28−123)	<.001
WBCs (10^9^/L)	8.6 (6.8−12.1)	8.1 (6.6−11.3)	11.77 (10.1−16.1)	.003
RBCs (10^12^/L)	4.0 ± 0.57	3.99 ± 0.52	4.01 ± 0.74	.646
Hb (g/L)	120.7 ± 16.5	120.2 ± 16.5	122.8 ± 17.0	.592
Platelet (10^9^/L)	239 ± 105	229 ± 80	272 ± 172	.122
NT‐ProBNP (pg/mL)	5264 (2559−10 294)	4555 (2142−8516)	10 410 (8043−21 015)	<.001
ALT (U/L)	36 (21−132)	34.5 (21.5−98.75)	122 (18−1944)	.123
AST (U/L)	44 (22−192)	41.5 (19.8−154.8)	177 (38−3026)	.007
Total bilirubin (μmol/L)	16.1 (11.4−24.6)	15.4 (11−22.15)	20.7 (12−55.6)	.063
Creatinine (μmol/L)	92 (77−129.5)	89.5 (75.8−109.3)	103.0 (82−252)	.144
Vasoactive drugs	56 (69.1%)	38 (61.3%)	18 (94.7%)	.006
MCS	43 (53.1%)	29 (46.8%)	14 (73.7%)	.040
Ventricular aneurysm	52 (64.2%)	42 (67.7%)	10 (52.6%)	.936
Coronary lesions				.193
Single‐vessel	34 (42.0%)	27 (43.5%)	7 (36.8%)	
Two‐vessel	43 (53.1%)	34 (54.8%)	10 (52.6%)	
Three‐vessel	4 (4.9%)	1 (1.6%)	2 (10.5%)	
Target vessel				.412
Anterior descending	65 (80.2%)	51 (82.3%)	14 (73.7%)	
Right coronary	16 (19.8%)	11 (17.7%)	5 (26.3%)	
Treatment timing^a^				.117
Emergency	17 (23.0%)	17 (29.8%)	2 (11.8%)	
Elective (before closure)	47 (63.5%)	31 (54.4%)	14 (82.4%)	
Elective (after closure)	10 (13.5%)	9 (15.8%)	1 (5.9%)	
Treatment approach				.812
PCI	56 (69.1%)	44 (71.0%)	12 (63.2%)	
PTCA	18 (22.2%)	13 (21.0%)	5 (26.3%)	
Conservative treatment	7 (8.6%)	5 (8.1%)	2 (10.5%)	
Defect site				.624
Apex	63 (77.8%)	49 (79%)	14 (73.7%)	
Posterior septum	18 (22.2%)	13 (21%)	5 (26.3%)	
Defect diameter (mm)	13.2 (10−16.1)	13 (9.45−16.0)	14.6 (10.0−20.0)	.142
Occluder diameter (mm)	26 (22−28)	26 (21.5−28)	26 (22−28)^b^	.251
T (d)	24 (20.5−31.5)	25.5 (22−34.25)	21 (18−23)	.004
Residual shunt (mm)	3.3 ± 2.0	3.1 ± 2.0	3.9 ± 2.1^b^	.245
Successful closure	76 (93.8%)	62 (100%)	14 (73.7%)	<.001

Abbreviations: ALT, alanine aminotransferase; AMI, acute myocardial infarction; AST, aspartate aminotransferase; CRP, C‐reactive protein; Hb, hemoglobin; HR, heart rate; LVEF, left ventricular ejection fraction; LViDd, left ventricular internal diameter at end‐diastole; MCS, mechanical circulatory support (intra‐aortic balloon pump counterpulsation [IABP] and extracorporeal membrane oxygenation [ECMO]); NT‐ProBNP, N‐terminal pro brain natriuretic peptide; PASP, pulmonary artery systolic pressure; PCI, percutaneous coronary intervention; PTCA, percutaneous transluminal coronary angioplasty; RBC, red blood cell; SBP, systolic blood pressure; T, time from AMI to VSR closure; WBC, white blood cell.

a *N* = 74, excluding 7 cases with conservative treatment.

b *N* = 14.

In a comparison of the death and survival groups, CRP, WBC, NT‐ProBNP, and AST were much higher in the death group (*p* < .01), and more patients in the death group received vasoactive drugs and mechanical circulatory support (MCS).

Compared to the survival group, the death group had a shorter time from AMI to VSR closure (*p* < .05), along with a lower success rate of the operation (*p* < .001). Among the VSR cases, culprit lesions were much more common in the anterior descending coronary artery, accounting for 80.2% of lesions, with the rest being in the right coronary arterial vessels. No patients with circumflex infarction were observed. All apical defects were caused by infarction of the anterior descending coronary artery, while most posterior septal defects were due to infarction of the right coronary artery. Only 2 posterior septal defects were caused by infarction of the anterior descending coronary artery (Table 1).

### 3.2 Primary safety outcomes and timing of closure

Among the 81 patients who underwent percutaneous VSR closure, there were 19 deaths (23.5%) at the 30‐day follow‐up, including 15 in‐hospital deaths and 4 out‐of‐hospital deaths. Five patients (6.2%) had failed occlusion, and all of these patients died in the hospital. Among these 5 patients, 2 patients were scheduled for additional surgical repair due to loose tissue surrounding the defect and gradual expansion and dislodgement of the occluder after release, 2 patients could not closure for failed anchoring of the occluder as a result of a very large defect size (>20 mm), and 1 case was managed with another surgical repair because the delivery sheath during the operation was folded, and pericardial tamponade developed due to free wall rupture during correction. Detailed information on the patients with failed occlusion is presented in Supporting Information: Table.

Primary endpoint outcomes were monitored after VSR closure performed at different time points. Since there were cases with unclear symptoms or delayed diagnosis of VSR on ultrasound, the diagnosis of VSR onset in this population is commonly not as accurate as the diagnosis of myocardial infarction. In this setting, we staged the time from AMI to VSR closure (T) as follows: T ≤ 2; 2 <T ≤ 3; 3 < T ≤ 4; and T > 4 weeks. The primary endpoint outcomes for VSR performed in different time periods are shown in Supporting Information: Figure 2. Patients who underwent VSR closure 3 weeks after infarction had better prognoses than within 3 weeks of infarction, accompanied by a lower mortality rate (7/56 [12.5%] vs. 12/25 [48%]; *p* < .001).

**Figure 1:**
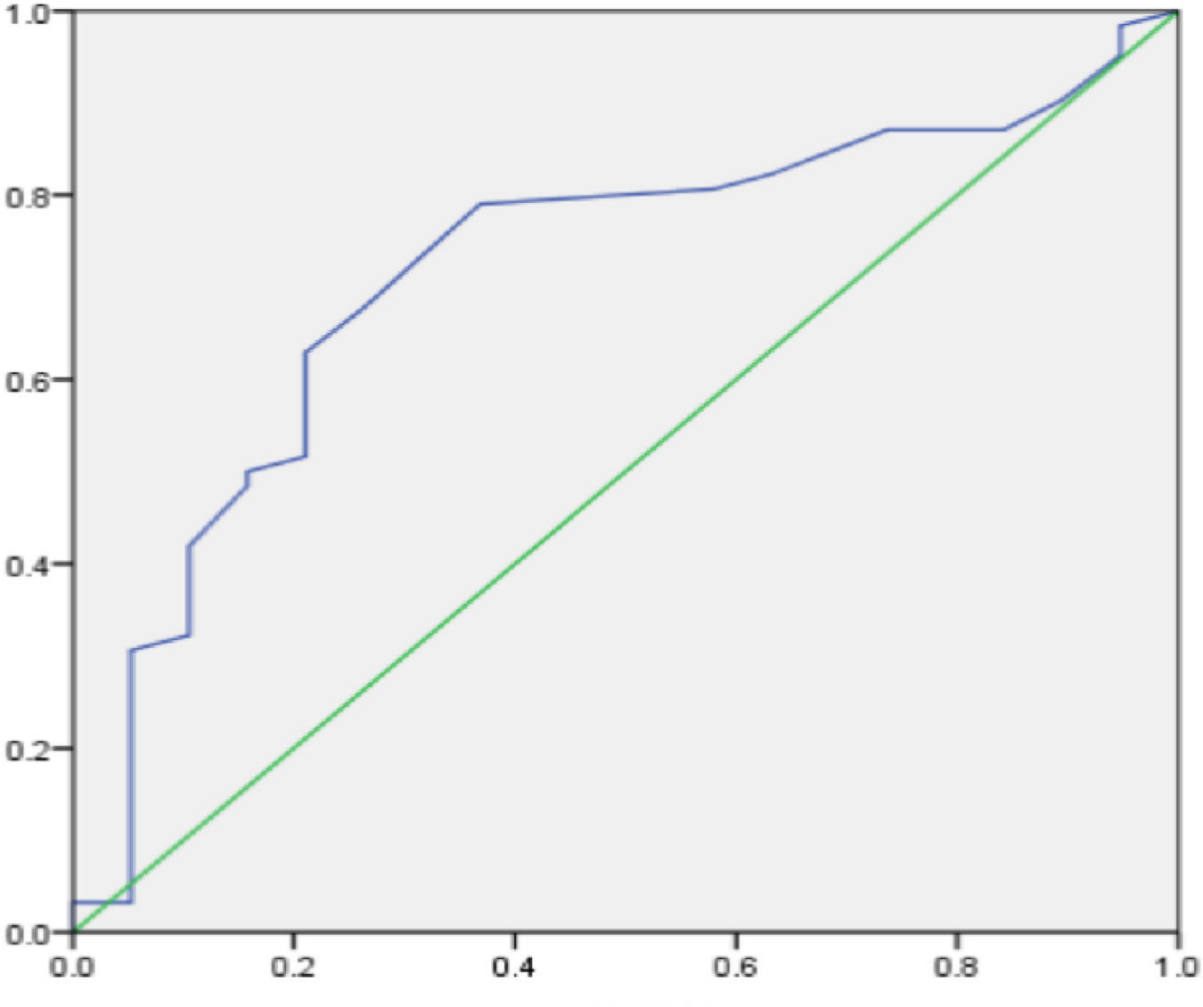
ROC curves showing the optimum time for VSR closure. VSR, ventricular septal rupture.

ROC curve analysis was performed to predict the optimum time for VSR closure, with T as the test variable and survival status at 30 days as the outcome variable. The results are shown in Figure 1. The area under the ROC curve was 0.720, with *p* = .004, which indicated statistical significance. The detection threshold was 21.5 days, the sensitivity was 0.790, and the specificity was 0.632.

### 3.3 Secondary safety outcomes

There were 76 patients with successful closure, among which 16 patients (21.1%) developed procedural complications, with valve damage in 12 patients (15.8%), vascular complications in 2 patients (2.6%), and hemolysis in 2 patients (2.6%). There was no high‐degree atrioventricular block. Patients who developed procedural complications had a relatively large defect diameter (14.8 [12.9−19.8] vs. 11.8 [8.7−16], *p* = .008) and required an occluder with a larger diameter (28 [26−28] vs. 24 [20−26], *p* = .005), with significant differences from patients without procedural complications. See Table 2 for details.

**Table 2:** Detailed information on patients with procedural complications.

Complications	Total cases	Posterior septal perforation case	Defect diameter (mm)	Occluder diameter (mm)	T (d)	Outcome
Valve damage	12					
MR/TR	3	2	16/20/16	28/28/26	171/50/16	1 out‐of‐hospital death, 2 survivors
TR	6	2	18/20/16/13/15/22	28/28/26/28/26/28	21/25/27/35/22/21	1 in‐hospital death after thoracotomy in the Surgery Department, 5 survivors
AR	1	1	15	26	16	Survivor after thoracotomy in the Surgery Department
MR	2	0	15/19	28/28	20/24	2 survivors after thoracotomy in the Surgery Department
Vascular damage	2	0	9/15	20/28	22/21	1 in‐hospital death, 1 out‐of‐hospital death
Hemolysis	2	0	20/14	28/26	13/16	1 in‐hospital death, 1 survivor

Abbreviations: AMI, acute myocardial infarction; AR, aortic regurgitation; MR, mitral regurgitation; T, time from AMI to VSR closure; TR, tricuspid regurgitation; VSR, ventricular septal rupture.

Nonprocedural complications: 1 patient (1.3%) had a MACE. The patient underwent interventional VSR closure and PCI in the same period and experienced recurrence of infarction on the day after the operation. He died because of circulatory collapse. One patient (1.3%) developed multiple organ failure after the operation and died in the hospital. Two patients (2.6%) had gastrointestinal bleeding, and both died in the hospital. No stroke was observed during the follow‐up.

### 3.4 Effectiveness outcomes

All 76 patients with successful closure underwent ultrasound after the operation. There were 66 patients who completed the cardiac function assessment at discharge, and there were 10 in‐hospital deaths. A total of 62 survivors were followed‐up by telephone interviews and assessed for cardiac function after 30 days (4 died outside the hospital). Cardiac function comparisons: Significant improvement of cardiac function was noted in patients at discharge and at 30 days after closure compared to before the operation (*p* < .001) (Supporting Information: Figure 3), while no evident difference was noted between patients at discharge and patients at 30 days after closure (*p* = .379). Cardiac catheterization and ultrasound indicators: Qp/Qs and PASP were significantly decreased after closure compared to before closure, whereas ASP was increased (*p* < .001). The LVEF and LViDd were also significantly improved after closure (*p* < .05). See Table 3 for details.

**Table 3:** Echocardiography indicators before and after VSR closure.

Echocardiography indicator	Before closure (*N* = 76)	After closure (*N* = 65)	*p* Value
Qp/Qs	3.05 ± 1.13	1.87 ± 0.49	<.001
PASP (mmHg)	54.8 ± 14.4	44.2 ± 13.6	<.001
ASP (mmHg)	108.3 ± 15.5	121.9 ± 19.0	<.001
LVEF value (%)	50.3 ± 8.0	52.1 ± 7.0	.014
LViDd (mm)	53.4 ± 5.9	51.9 ± 6.6	.029

Abbreviations: ASP, aortic systolic pressure; LVEF, left ventricular ejection fraction; LViDd, left ventricular internal diameter at end‐diastole; PASP, pulmonary artery systolic pressure; Qp/Qs, pulmonary/systemic flow ratio.

### 3.5 Multivariate regression analysis of all‐cause mortality at 30 days after closure

Indicators that showed statistically significant differences for the survival group versus the death group, including CRP, WBC, NT‐ProBNP, AST, application of vasoactive drugs, and T, were included in multivariate analysis. In addition, prognostic significance, closure failure, and procedural complications were included to analyze their effect on mortality at 30 days after closure. The threshold for continuous variables was determined by ROC analysis. The multivariable analysis showed that CRP levels greater than 26 mg/L was significantly associated with 30‐day mortality (OR: 74.72, 95% CI: 2.40−2323.35, *p*: .014). Another significant predictors of 30‐day mortality was NT‐ProBNP greater than 6000 pg/mL (OR: 178, 95% CI: 3.19−9931.83, *p*: .012). Other indicators have no significant impact on 30‐day mortality rate. See Table 4 for details.

**Table 4:** Multivariate logistic regression analysis of the risk factors for 30‐day mortality after closure.

Variables	OR (95% CI)	*p* Value
CRP (mg/L)		
≤26	1.00	
>26	74.72 (2.40−2323.35)	.014
WBC (10^9^/L)		
≤9.8	1.00	
>9.8	7.87 (0.65−95.03)	.105
NT‐ProBNP (pg/mL)		
≤6000	1.00	
>6000	178.00 (3.19−9931.83)	.012
AST		
≤85	1.00	
>85	4.82 (0.38−61.68)	.226
Vasoactive drugs		
No	1.00	
Yes	4.14 (0.12−141.64)	.431
T (d)		
>21	1.00	
≤21	9.00 (0.80−101.47)	.075
Closure failure or procedural complications		
No	1.00	
Yes	6.79 (0.44−104.59)	.170

Abbreviations: AMI, acute myocardial infarction; AST, aspartate aminotransferase; CRP, C‐reactive protein; NT‐ProBNP, N‐terminal pro brain natriuretic peptide; T, time from AMI to VSR closure; VSR, ventricular septal rupture; WBC, white blood cell.

## 4 DISCUSSION

VSR is one of the most severe mechanical complications after AMI. Although thrombolysis or emergency PCI after AMI can contribute to a decreased incidence of VSR, there is still an exceedingly high mortality rate after VSR occurs. In the absence of active treatment, approximately 60%−70% of patients will die within 2 weeks after VSR, and no more than 10% will survive for 3 months.^8^ Sánchez Vega JD et al.^9^ statistically analyzed 120 VSR patients from 11 tertiary hospitals in Spain and noticed that the in‐hospital mortality rate was 60% and the 1‐year mortality rate was up to 61.6%.

In recent years, MCS techniques, such as intra‐aortic balloon pump counterpulsation (IABP) and extracorporeal membrane oxygenation, have been gradually applied in an increasing number of medical centers. More VSR patients are able to achieve hemodynamic stability and the opportunity to undergo closure treatment in later stages.^10^ With the development of interventional therapeutic techniques, interventional closure has become an option for an increasing number of patients with VSR. Regarding the effectiveness and safety of the interventional strategy, relevant studies have been conducted in our center and other medical centers. However, these studies were based on a small sample size, and large‐scale clinical trials are required for further analysis. In 2013, Assenza et al.^11^ investigated 30 AMI cases complicated with ASR; 12 patients underwent direct VSR closure, with an average closure time of 19 days and a 30‐day mortality rate of 42%, while the other 18 patients received interventional closure for a residual shunt after surgery, with an average closure time of 54 days and a 30‐day mortality rate of 11%. A meta‐analysis performed by Risseeuw et al.^5^ revealed that patients who underwent interventional VSR closure in the acute phase (<14 days) had a mortality rate of 46%−100%, significantly higher than the 20%−38.9% mortality rate in patients who underwent closure in the chronic phase (>14 days). Egbe et al.^12^ conducted a clinical trial of patients who underwent interventional VSR closure at the Mayo Clinic and reported survival rates of 70% and 61% at 1 month and 5 years, respectively. Calvert et al.^13^ studied 53 cases of VSR managed by interventional closure and reported 48 successful cases (92%); 18 patients died before discharge, with 31 patients surviving to discharge.

The current work reported 81 patients who underwent interventional closure treatment for VSR at our center, including 76 successful cases (93.8%), 15 in‐hospital deaths (18.5%), and 19 deaths at 30 days (23.5%). Currently, there is controversy about the optimal timing for the closure of VSR. Consistent with previous reports, we found that the 30‐day mortality rate of interventional VSR closure was better when performed 3 weeks after infarction compared to closure within 3 weeks of infarction (12.5% vs. 48%, *p* < .001). Additionally, we utilized the ROC curve method for the first time to calculate the optimal timing for VSR closure, which was found to be 21 days after MI. However, we only enrolled VSR patients who underwent operation, and those who died in the acute phase that were unable to receive interventional treatment were excluded, which may have resulted in selection bias.

The occluder should be selected according to the principal “large rather than small.” Even if operated after 3 weeks, there is still some myocardial tissue surrounding the defect that is not completely “hardened.” This might explain that the 2 patients who failed occlusion at our center experienced dislodgement of the occluder after released. In addition, the defect was generally irregular in shape, largely characterized by multichannel and sieve‐like features, which are likely to cause residual shunts. In this context, an occluder with a large size is recommended to fully close the defect as much as possible to reduce the incidence of residual shunts. We used an occluder with a diameter 8−14 mm greater than the defect diameter during the operation and achieved good results.

Procedural complications are known to affect patient prognosis. We found that patients who developed procedural complications suffered a relatively high mortality rate at 30 days of closure compared to that of patients without complications, but this difference was not significant (5/16 [31.3%] vs. 9/60 [15%], *p* = .136). We further found that these complications were mainly valve damage (12/16), along with a higher proportion of posterior septal perforation (5/12 [41.7%] vs. 11/64 [17.2%], *p* = .056), which might be associated with the adjoined posterior septum and mitral or tricuspid valve. In the case of posterior septal perforation and the application of a larger occluder, the delivery system and the occluder easily rupture the chordae tendineae, leading to valve damage. As shown in Supporting Information: Figure 4, patients with infarction tended to have chordae tendineae and papillary muscle ischemia and even necrosis, with the tissue becoming fragile and susceptible to damage. Therefore, an occluder of an appropriate size should be selected if closure is performed for posterior septal perforation, and gentle handling during operation is needed to avoid valve damage. Beyond procedural complications, infection and poor cardiac function are factors with great implications for patient prognosis. In this multivariate regression analysis of patient 30‐day mortality after closure, increases in CRP and NT‐ProBNP were risk factors for early death. Hence, active infection prevention and correction of cardiac function should be implemented in VSR patients, and if necessary, MCS techniques such as IABP could be considered before hemodynamic instability events. Our previous research proved that IAPB is effective in improving hemodynamic stability in VSR patients and reducing left‐to‐right shunting.^14^

The goal of percutaneous closure is to reduce the left‐to‐right shunt and improve cardiac function. The present work proved the effectiveness of interventional VSR closure in improving cardiac function. Cardiac catheterization and echocardiography data revealed significant reductions in Qp/Qs and PASP after closure and an evident increase in ASP. In addition, LVEF and LViDd improved to some extent.

The treatments for VSR mainly include conservative treatment using drugs, interventional closure and surgery. The present study focused on the interventional strategy, but there are no relevant studies comparing this approach with drug therapy or surgery, which is a limitation of the study. Additionally, the mechanism of VSR after AMI has rarely been reported. Liu et al.^15^ identified multiple risk factors for VSR after AMI, including advanced age, AMI recurrence in the hospital, low systolic blood pressure, lesions in the left anterior descending artery, decreased hemoglobin, low total protein, and high serum magnesium, but further clinical trials are required.

In conclusion, VSR is a life‐threatening condition associated with high mortality rates. Our study suggests that percutaneous VSR closure can be a valuable treatment option for suitable patients with VSR after AMI, with the optimal closure time being 3 weeks after AMI. However, increases in CRP and NT‐ProBNP should be monitored closely as they are associated with higher risk of death at 30 days after closure.

## CONFLICT OF INTEREST STATEMENT

The authors declare no conflict of interest.

## Supporting information

Supporting information.

## Data Availability

The data that support the findings of this study are available from the corresponding author, [Chuanyu Gao], upon reasonable request.
