# Supplementary material for: Percutaneous closure of ventricular septal rupture after myocardial infarction: A retrospective study of 81 cases
Source: Clin Cardiol. 2023 May 15;46(7):737–44. doi: 10.1002/clc.24027 (PMC10352964; doi:10.1002/clc.24027)
Supplement: Supplementary file 1 — Supporting information. [file CLC-46-737-s001.docx]

**S. Table Detailed information on patients with failed VSR closure**

| Number | Gender | Age | Culprit vessel | Defect site | Defect diameter (mm) | Occluder diameter (mm) | T (Days) | Causes for failure | Outcome |
| --- | --- | --- | --- | --- | --- | --- | --- | --- | --- |
| 1  2  3  4  5 | Male  Female  Male  Female Male | 67  62  65  71  73 | Anterior descending Anterior descending  Right coronary Anterior descending Right coronary | Apex  Apex  Posterior septum  Apex  Posterior septum | 14  16  20  10  22 | 26  26  28  28  28 | 20  25  21  22  16 | Occluder dislodgement  Occluder dislodgement  Large defect  Pericardial tamponade  Large defect | In-hospital death  In-hospital death  In-hospital death  In-hospital death  In-hospital death |

T: time from AMI to VSR closure.


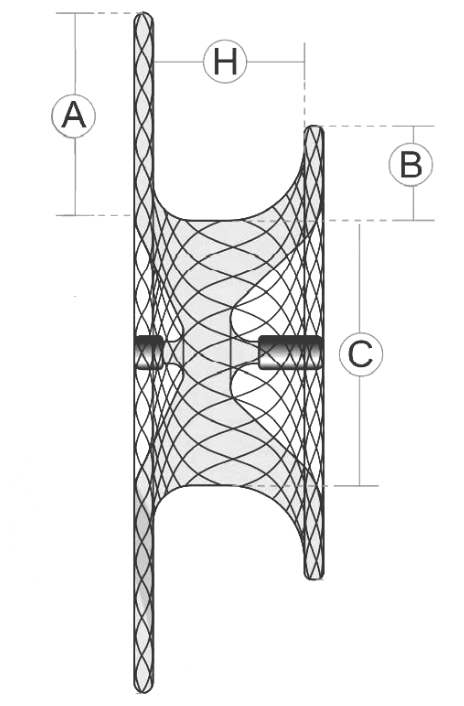


**S. Figure 1 The closure device with a 7 mm left ventricular side rim (A), a 3 mm right ventricular side rim (B) and a 10 mm waist height (H).**

**S.Figure 2 Outcomes of VSR closure in different time periods**


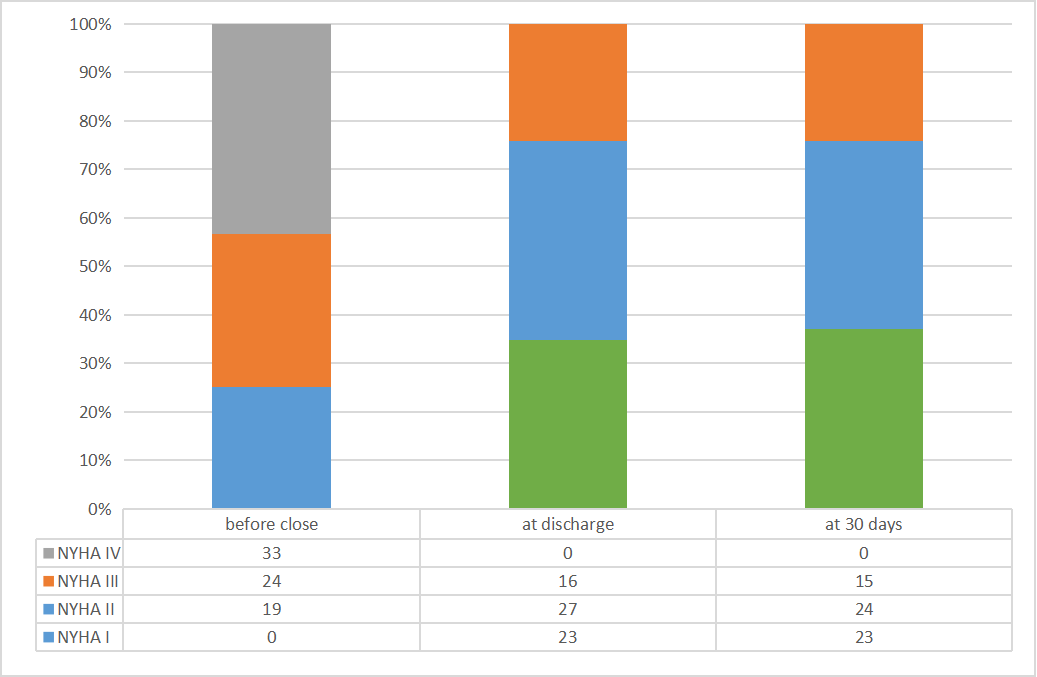


**S. Figure 3 NHYA Cardiac function before close, at discharge and at the 30-day follow-up**


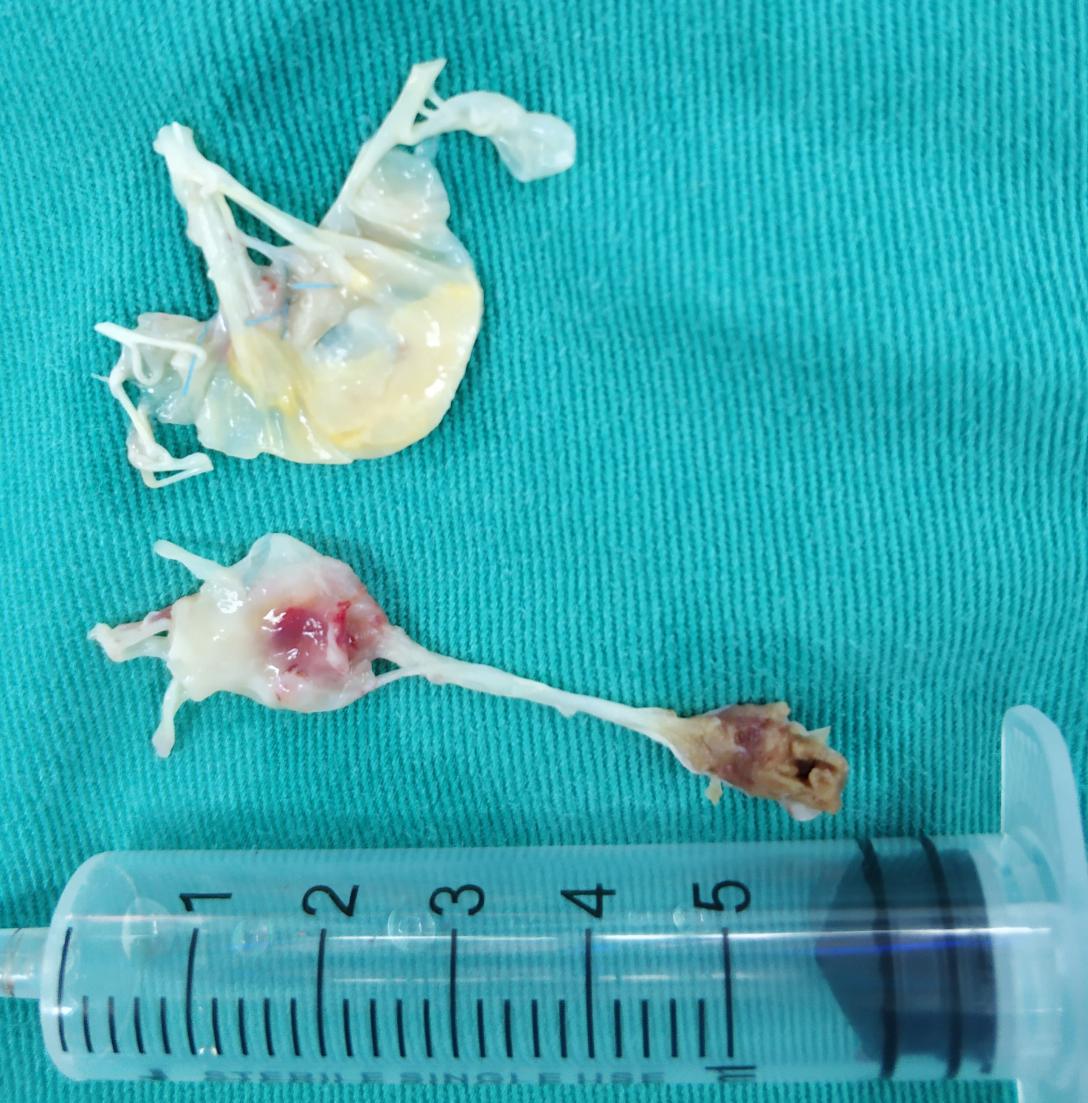


**S.Figure 4 Anterior mitral valve chordae tendineae and papillary muscle injured during occlusion due to ischemia after MI**
